# Clinical, Histopathological, and Radiological Profile of Patients Presenting With Thyroid Malignancies Among the Kerala Population

**DOI:** 10.7759/cureus.56775

**Published:** 2024-03-23

**Authors:** Chandralal Prasannakumar Girijakumari, Arshad Koroth, Muhamed Fawas Abdul Rasheed, Azif Ali Usman, Shiraz Basheer, Mohamed Rasween Kareem

**Affiliations:** 1 Department of Surgical Oncology, All India Institute of Medical Sciences, Jodhpur, Jodhpur, IND; 2 Department of General Surgery, Kasturba Medical College, Manipal, Manipal, IND; 3 Department of General Surgery, Muslim Educational Society (MES) Academy Of Medical Sciences, Perinthalmanna, IND; 4 Department of General Surgery, Muslim Educational Society (MES) Medical College, Perinthalmanna, IND

**Keywords:** thyroid malignancies, papillary carcinoma, microcalcification, follicular carcinoma, echogenicity

## Abstract

Background and objective: Thyroid cancer, though relatively uncommon among all cancer types, stands as the primary form of endocrine tumor. Over the last 20 years, there has been a significant uptick in its occurrence. Papillary thyroid carcinoma (PTC), which is well-differentiated, emerges as the dominant subtype, in regions where iodine levels are deemed adequate. The study aimed to study the clinicopathological profile of patients diagnosed with thyroid malignancies at the Muslim Educational Society (MES) Medical College Perinthalmanna.

Materials and methods: This is a retrospective study undertaken at the MES Medical College by the Department of General Surgery and Endocrine Surgery. The study focuses on patients who have been diagnosed with thyroid cancer through a biopsy. Case sheets of all those patients diagnosed with thyroid malignancy were referred from the Medical Records Library to collect the relevant medical and sociodemographic data. This data was entered in the proforma, which was transferred to the Excel sheet and processed in IBM SPSS Statistics for Windows, Version 20 (Released 2011; IBM Corp., Armonk, New York, United States).

Results: The study included predominantly middle-aged individuals (40-60 years), with 22 (55%) falling within this age range, followed by 14 (35%) aged between 20 and 40 years, and only four (10%) above 60 years. Female patients constituted 82.5% of the study group. Most cases presented with swelling lasting less than six months 23 (57.5%), while only four (10%) had swelling lasting more than five years. Compression symptoms were rare, with only three (7.5%) experiencing dysphagia or dyspnea. Pain was reported in two (5%) of the cases. Hypothyroidism, toxic manifestations, or hoarseness were observed in one (2.5%) of the patients. Regarding swelling characteristics, most were greater than 4 cm in size (29, 72%) and firm in consistency (25, 62.5%). Nodular surfaces were present in 19 (47.5%) of the cases, while 38 (95%) of the swellings were mobile. Palpable lymph nodes were noted in 13 (32.5%) of cases. Radiologically, hypoechoic lesions were observed in 26 (65%) of cases, with microcalcification in 29 (72.5%) and peripheral vascularity in 31 (77.5%). Papillary carcinoma was the most common histological type (34, 85%), with medullary and follicular carcinomas accounting for five (12.5%) and one (2.5%), respectively. An association was found between the duration of swelling and histological type (p = 0.05) and between the mobility of swelling and histological type (p < 0.05). However, no significant associations were observed between imaging findings and histological type (p > 0.05). The gender distribution did not show a statistically significant association with histological type.

Conclusion: The findings of the study revealed a statistically insignificant association between age, gender, clinical features, and the histological type of thyroid malignancy. Additionally, there was no statistically significant association between the histological type of thyroid malignancy and the size or type of surface or consistency of thyroid swelling or ultrasonographic findings of the swelling like echogenicity, microcalcification, increased peripheral vascularity, or loss of peripheral halo.

## Introduction

Thyroid cancer constitutes a mere 1% of all malignancies, although it is the prevailing type of endocrine tumor. The occurrence of thyroid cancer has risen by a factor of four in females and three in males during the last 20 years [1]. The prevalence of palpable thyroid nodules in adults ranges from 4% to 7%. The frequency significantly increases when considering the inclusion of nodules identified using ultrasonography or discovered at autopsy [2]. It was also documented that almost 50% of individuals who are 60 years old have thyroid nodules [3]. Approximately 5% to 20% of nodules that are noticed during clinical examination are found to be malignant [4]. Well-differentiated thyroid cancer (WDTC), specifically papillary thyroid carcinoma (PTC), is the predominant form of thyroid malignancy, accounting for around 90% of newly diagnosed cases in regions with acceptable iodine levels. Among the reported cases, Asian women had the highest occurrence of WDTC, with PTC being the most prevalent kind [5].

While thyroid nodules are prevalent, only a small number are cancerous and necessitate surgical intervention. It is crucial to employ an organized strategy in assessing them to prevent unwarranted surgical procedures [6]. Thyroid gland cancer arises from either the thyroid follicular (acinar) cells or the parafollicular (C) cells. The predominant types of tumors are epithelial and arise from the thyroid follicular cells. These include papillary carcinoma, follicular carcinoma, Hurthle cell carcinoma, poorly differentiated carcinoma, and anaplastic (undifferentiated) carcinoma [7]. Medullary thyroid carcinoma (MTC) is an uncommon malignant tumor that originates from the C cells of the thyroid gland. Most cases of thyroid tumors are classified as differentiated thyroid carcinomas, specifically papillary or follicular carcinomas, which have a high likelihood of being cured and show a favorable response to radiation therapy [8-10]. Due to its invasive nature, MTC necessitates careful monitoring and comprehensive interdisciplinary treatment. The clinical manifestation, treatment, and result of MTC varied among various groups [10]. MTC is an uncommon kind of thyroid cancer that arises from C cells. It represents 5% of all thyroid carcinomas and has a disease-specific death rate of up to 13% [9].

The most common variety is the papillary subtype, which comprises 70%-80% of all thyroid cancers. It has been observed that there is a wide difference in the prevalence of thyroid malignancies based on geographical location. Low rates are observed in certain European countries and high rates in Hawaii. Along similar lines, the incidence of this disease varies uniquely with age, sex, and ethnicity [11]. The overall prognosis of WDTC is often excellent, with survival rates surpassing 90% after 10 years [12]. Nevertheless, several patient subgroups have exhibited an increased risk of recurrence and mortality [13]. This leads to the requirement that each thyroid nodule be screened for thyroid cancer. Given the increased longevity of individuals with thyroid cancer, it is crucial to accurately describe their clinical characteristics and gain an in-depth comprehension of the progression of the disease and its clinical outcomes. However, research is scarce in our specific context that assesses the prevalence of thyroid nodules upon initial presentation and elucidates the features that are related to thyroid cancer [14]. Hence, the study aimed to evaluate the clinicohistopathological and radiological profiles of patients diagnosed with thyroid malignancies at Muslim Educational Society (MES) Medical College Perinthalmanna.

## Materials and methods

This study was conducted in a hospital setting and involved a retrospective analysis of patients who were diagnosed with thyroid cancer through a biopsy. All patients, irrespective of age, with biopsy-proven thyroid malignancies were included. Those who were not willing to participate and had incomplete medical records were excluded. Case sheets of all those patients diagnosed with thyroid malignancy were referred from the Medical Records Library to collect the relevant medical and sociodemographic data.

The Institutional Ethics Committee of MES Medical College accorded ethical approval (IEC/MES/34/2019). The sample size was calculated based on an earlier prevalence estimate of 89% [15], employing the formula: N = Z^2^p(1−p)d^2^. In this formula, Z represents the confidence level of 95% (with a standard value of 1.96), d represents the allowable error of 5%, p represents the estimated prevalence from a comparable investigation, and d represents the allowable error of 10%. The minimum estimated sample size was 40 patients with biopsy-proven thyroid malignancies at MES Medical College under the Department of General Surgery and Endocrine Surgery between January 2018 and December 2019.

Statistical analysis

The information gathered was entered and analyzed with IBM SPSS Statistics for Windows, Version 20 (Released 2011; IBM Corp., Armonk, New York, United States). The qualitative variables were expressed as frequencies and percentages and were compared using either the Chi-square or Fisher's exact tests. The threshold for statistical significance was established at a p-value of ≤0.05.

## Results

Table 1 presents the demographic and clinical characteristics of the study population. The majority of participants (55%; n = 22) were aged between 40 and 60 years, followed by 35% (n = 14) in the age group of 20 to 40 years. Only 10% (n = 4) of the participants were aged above 60 years. In terms of gender distribution, the sample comprised 82% (n = 33) females and 18% (n = 7) males. Regarding the duration of the swelling, 57.5% (n = 23) of cases had the swelling for less than six months, while 17.5% (n = 7) had the swelling for 1-5 years. There were only 10% (n = 4) of cases with a swelling duration exceeding five years. All participants presented with a swelling, and the size of the swelling was assessed. It was found that 72% (n = 29) had a swelling size greater than 4 cm, 23% (n = 9) had a swelling size between 2 and 4 cm, and only 5% (n = 2) had a swelling size less than 2 cm.

**Table 1:** Distribution of age, gender, duration, and size of the swelling among the study population

Variables		Frequency (n)	Percent (%)
Age group	20-40	14	35.0
	40-60	22	55.0
	>60	4	10.0
	Total	40	100.0
Gender	Males	7	18
	Females	33	82
Duration	<6 months	23	57.5
	6-12 months	6	15.0
	1-5 years	7	17.5
	>5 years	4	10.0
	Total	40	100.0
Size of the swelling	<2 cm	2	5.0
	2-4 cm	29	72.5
	>4 cm	9	22.5
	Total	40	100.0

The study revealed that all cases within the study population exhibited neck swelling. Only 7.5% (n = 3) of cases displayed symptoms of compression, such as dysphagia or dyspnea, while 5% (n = 2) experienced pain. The occurrence of hoarseness was observed in only 2.5% (n = 1) of cases. Among the cases examined, 47.5% (n = 19) had nodular surfaces, whereas 52.5% (n = 21) had smooth surfaces. The lower border of the swelling was palpable in all cases except one. Regarding consistency, 62.5% (n = 25) of the swellings were firm, 27.5% (n = 11) were hard, and only 10% (n = 4) were soft. Additionally, 95.0% (n = 38) of the swellings were found to be mobile, while only 32.5% (n = 13) of cases exhibited palpable lymph nodes (Table 2).

**Table 2:** Clinical presentation of symptoms among the study population N: Number of cases; %: percentage

Main symptoms		N (%)
Neck swelling		40 (100%)
Pain		2 (5%)
Dysphagia/dyspnea		3 (7.5%)
Hoarseness		1 (2.5%)
Signs in cases of solitary thyroid nodules (n = 40)		
Surface	Nodular	19 (47.5%)
	Smooth	21 (52.5%)
Lower border	Palpable	39 (97.5%)
	Not palpable	1 (2.5%)
Consistency	Soft	4 (10%)
	Firm	25 (62.5%)
	Hard	11 (27.5%)
Mobility	Yes	38 (95.0%)
	No	2 (5%)
Lymph nodes	Yes	13 (32.5%)
	No	27 (67.5%)

Among the sample population, the majority, 65% (n = 26), exhibited hypoechogenicity. Additionally, 72.5% (n = 29) of cases displayed microcalcification, while 77.5% (n = 31) showed peripheral vascularity. In terms of peripheral halo, 52.5% (n = 21) of cases did not exhibit any loss, while 47.5% (n = 19) experienced a loss of peripheral halo (Table 3).

**Table 3:** Frequency of echogenicity among the cases

Variables		Frequency (N)	Percentage (%)
Echogenicity	Hypo	26	65.0
	Hyper	4	10.0
	Iso	1	2.5
	Mixed	9	22.5
	Total	40	100.0
Microcalcification	Yes	29	72.5
	No	11	27.5
	Total	40	100.0
Peripheral vascularity	Yes	31	77.5
	No	9	22.5
	Total	40	100.0
Loss of peripheral halo	Yes	19	47.5
	No	21	52.5
	Total	40	100.0

The Chi-square test showed no significant association (p = 0.600) between age groups and histological types of carcinomas, indicating that age groups were comparable in terms of histological types (Table 4).

**Table 4:** Comparison of the histological types of carcinomas and age group N: Number of cases; %: percentage p-value was considered significant if <0.05.

Variables		Histological types			Total, N (%)	p-value
		Follicular carcinoma, N (%)	Medullary carcinoma, N (%)	Papillary carcinoma, N (%)		
Age	20-40	1 (7.14%)	1 (7.14%)	12 (85.72%)	14 (100%)	0.600
	40-60	0 (0%)	3 (13.64%)	19 (86.36%)	22 (100%)	
	>60	0 (0%)	1 (25%)	3 (75%)	4 (100%)	
Total		1 (2.5%)	5 (12.5%)	34 (85.0%)	40 (100%)	

In the 40-60 age group, 86.36% (n = 19) had papillary carcinoma, while in the 20-40 age group and >60 age group, 85.72% (n = 12) and 75% (n = 3), respectively, had the same condition. Regarding gender distribution, 84.85% (n = 28) of females and 85.7% (n = 6) of males were diagnosed with papillary carcinoma (Table 5).

**Table 5:** Comparison of the histological types of carcinomas with gender N: Number of cases; %: percentage p-value was considered significant if <0.05.

Variables		Histological types			Total, N (%)	p-value
		Follicular carcinoma, N (%)	Medullary carcinoma, N (%)	Papillary carcinoma, N (%)		
Gender	Female	1 (3.03%)	4 (12.12%)	28 (84.85%)	33 (100%)	0.889
	Male	0 (0%)	1 (14.3%)	6 (85.7%)	7 (100%)	
Total		1 (2.5%)	5 (12.5%)	34 (85.0%)	40 (100%)	

The current study also assessed the association between gender groups and histological types using a Chi-square test. It was also found to be comparable as the p-value was 0.889 (p > 0.05), which was statistically insignificant. The duration of the swelling was classified as less than six months, 6-12 months, 1-5 years, and more than five years. When it was compared with the histological types of the swelling, it was found to be significant (p = 0.05). It was noted that the majority of papillary carcinoma types had a swelling duration of less than six months (Table 6).

**Table 6:** Comparison of the histological types of carcinomas with the duration of the swelling N: Number of cases; %: percentage p-value was considered significant if <0.05.

Variables		Histological types			Total, N (%)	p-value
		Follicular carcinoma, N (%)	Medullary carcinoma, N (%)	Papillary carcinoma, N (%)		
Duration of the swelling	<6 months	0 (0%)	3 (13%)	20 (87.00%)	23 (100.0%)	0.05
	6-12 months	1 (16.67%)	0 (0.0%)	5 (83.33%)	6 (100.0%)	
	1-5 years	0 (0.0%)	0 (0.0%)	7 (100.0%)	7 (100.0%)	
	>5 years	0 (0.0%)	2 (50.00%)	2 (50.00%)	4 (100.0%)	
Total		1 (2.5%)	5`(12.5%)	34 (85.0%)	40 (100%)	

The study compared the different symptoms with histological types. The symptoms include pain, pressure effect, hoarseness, thyrotoxicosis, and hypothyroidism. There was a statistically insignificant association between the histological type of the swelling and symptoms as the p-value was found to be greater than 0.05 in all instances (Table 7).

**Table 7:** Comparison of the histological types of carcinomas with the symptoms N: Number of cases; %: percentage p-value was considered significant if <0.05.

Variables		Histological types			Total, N (%)	p-value
		Follicular carcinoma, N (%)	Medullary carcinoma, N (%)	Papillary carcinoma, N (%)		
Pain	Yes	0 (0.0%)	0 (0.0%)	2 (100.0%)	2 (100.0%)	0.830
	No	1 (2.63%)	5 (15.62%)	32 (84.2%)	38 (100.0%)	
Pressure effect	No	1 (2.7 %)	5 (13.5%)	31 (83.79%)	37 (100.0%)	0.966
	Dyspnea	0 (0.0%)	0 (0.0%)	1 (100.0%)	1 (100.0%)	
	Dysphagia	0 (0.0%)	0 (0.0%)	2 (100.0%)	2 (100.0%)	
Hoarseness of voice	Yes	0	0	1 (100.0%)	1 (100.0%)	0.913
	No	1 (2.56%)	5 (12.82%)	33 (84.61%)	39 (100.0%)	
Symptoms of thyrotoxicosis	Yes	0 (0.0%)	0 (0.0%)	1 (100.0%)	1 (100.0%)	0.913
	No	1 (2.56%)	5 (12.82%)	33 (84.61%)	39 (100.0%)	
Hypothyroidism	Yes	0 (0.0%)	0 (0.0%)	2 (100.0%)	2 (100.0%)	0.830
	No	1 (2.63%)	5 (13.15%)	32 (84.2%)	38(100.0%)	

Most of the swellings were of the size between 2 and 4 cm, and among that, majority belonged to the papillary carcinoma group (Table 8).

**Table 8:** Comparison of the histological types of carcinomas with the size of the swelling N: Number of cases; %: percentage p-value was considered significant if <0.05.

Variables		Histological types			Total, N (%)	p-value
		Follicular carcinoma, N (%)	Medullary carcinoma, N (%)	Papillary carcinoma, N (%)		
Size	<2 cm	0 (0.0%)	0 (0.0%)	2	2 (100.0%)	0.246
	2-4 cm	0 (0.0%)	5 (17.24%)	24 (82.76%)	29 (100.0%)	
	>4 cm	1 (11.1%)	0 (0.0%)	8 (88.9%)	9 (100.0%)	
Total		1 (2.5%)	5 (12.5%)	34 (85.0%)	40 (100%)	

The swellings that were greater than 4 cm majorly belonged to the papillary carcinoma group. However, these results did not have any statistical significance when it was tested with a Chi-square test (p > 0.05). The study couldn’t establish any statistically significant association between signs such as surface, palpability of the lower border, consistency of the swelling, or the presence of lymph nodes with the histological type as the Chi-square tests yield results with nonsignificant p-values (p > 0.05) (Table 9).

**Table 9:** Comparison of the histological types of carcinomas with signs N: Number of cases; %: percentage p-value was considered significant if <0.05.

Variables		Histological types			Total, N (%)	p-value
		Follicular carcinoma, N (%)	Medullary carcinoma, N (%)	Papillary carcinoma, N (%)		
Surface	Nodular	0 (0.0%)	3 (15.7%)	16 (84.3%)	19 (100.0%)	0.543
	Smooth	1 (4.76%)	2 (9.52%)	18 (85.71%)	21 (100.0%)	
Lower border	Palpable	1 (2.56%)	5 (12.82)	33 (84.62)	39 (100.0%)	0.913
	Not palpable	0 (0.0%)	0 (0.0%)	1 (100.0%)	1 (100.0%)	
Consistency	Soft	0 (0.0%)	0 (0.0%)	4 (100.0%)	4 (100.0%)	0.441
	Firm	1 (4.5%)	2 (8.00%)	22 (87.5%)	25 (100.0%)	
	Hard	0 (0.0%)	3 (27.27%)	8 (72.73)	11 (100.0%)	
Mobility	Yes	1 (2.6%)	3 (7.9%)	34 (89.5%)	38 (100.0%)	0.001
	No	0 (0.0%)	2 (100.0%)	0 (0.0%)	2 (100.0%)	
Lymph nodes	Yes	1 (7.7%)	3 (23.07%)	9 (69.29%)	13 (100.0%)	0.113
	No	0 (0.0%)	2 (7.4%)	25 (92.3%)	27 (100.0%)	

However, the study showed a significant association between the mobility of the swelling and the histological type (p = 0.001). Most of the cases in the study were hypoechoic lesions, and among these, most belonged to the papillary carcinoma group. However, this result did not have any statistical significance. There was not any microcalcification in the follicular carcinoma group, whereas it was present among medullary and papillary carcinomas (Table 10).

**Table 10:** Comparison of the histological types of carcinomas with imaging findings N: Number of cases; %: percentage p-value was considered significant if <0.05.

Variables		Histological types			Total, N (%)	p-value
		Follicular carcinoma, N (%)	Medullary carcinoma, N (%)	Papillary carcinoma, N (%)		
Echogenicity	Hypo	0 (0.0%)	4 (15.4)	22 (84.6%)	26 (100.0%)	0.487
	Hyper	0 (0.0%)	1 (25.0%)	3 (75.0%)	4 (100.0%)	
	Iso	0 (0.0%)	0 (0.0%)	1 (00.0%)	1 (100.0%)	
	Mixed	1 (11.1%)	0 (0.0%)	8 (88.9%)	9 (100.0%)	
Microcalcification	Yes	0 (0.0%)	4 (13.8%)	25 (86.2%)	29 (100.0%)	0.247
	No	1 (9.1%)	1 (9.1%)	9 (81.8%)	11 (100.0%)	
Increased peripheral vascularity	Yes	1 (3.2%)	2 (6.45%)	28 (90.35%)	31 (100.0%)	0.092
	No	0 (0.0%)	3 (33.3%)	6 (66.7%)	9 (100.0%)	
Loss of peripheral halo	Yes	0 (0.0%)	2 (10.5%)	17 (89.5%)	19 (100.0%)	0.576
	No	1 (4.76%)	3 (14.28%)	17 (80.96%)	21 (100.0%)	

There was a similar observation in the case of the peripheral halo. However, the study could not establish a statistically significant association between any of the imaging findings and the histological types (p > 0.05).

## Discussion

This retrospective study was done to assess the clinicopathological and radiological profiles of patients diagnosed with thyroid malignancies at MES Medical College Perinthalmanna. This study comprised a total of 40 participants. The study consisted primarily of participants aged 40-60 years, including 22 (55%) of the total sample. The second largest age group was individuals aged 20-40 years, accounting for 14 (35%) of the participants. This study revealed a predominance of females, with 82.5% of the study population being female. Prasanth et al. performed a study that showed a comparable distribution of genders [16]. In their study, 87.5% of the participants were females, and the rest were males [16]. More than half (23, 57.5%) of the study participants in this study reported the duration of their thyroid swelling as less than six months. About 15% had the swelling for 6-12 months, and 7 (17.5%) had the swelling for 1-5 years. Only 4 (10%) of the participants in this study reported that they had the swelling for more than five years. Melak et al. reported that the mean duration at the point of diagnosis was 5.32 years, ranging from one week to 50 years in their study [17].

In this study, only two (5%) of the participants reported having pain, while 38 (95%) of the participants didn’t have pain. The majority of the participants in this study had no pressure effects due to the swelling. Two (5%) of the participants reported having dysphagia, and one (2.5%) had dyspnea. One (2.5%) of the participants reported having hoarseness of voice. The study by Prasanth et al. on clinical profiles in cases of solitary thyroid nodules who were hospitalized in tertiary care hospitals reported that none of their participants had pain or pressure effects [16]. In their study, only one participant (2.5%) had symptoms of thyrotoxicosis, and two (5%) had symptoms of hypothyroidism. Further, none of their participants had toxic manifestations. Nayci et al. conducted a study in which they examined the relationship between hormone activity and the incidence of cancer [18]. Of the 351 patients with hyperthyroidism, 122 (34.8%) had malignant thyroid illness, whereas the other patients (65.2%) had benign diseases. A total of 849 patients were diagnosed with both hypothyroidism and euthyroidism. Out of the total, 212 individuals (25%) were diagnosed with malignant conditions, while 637 individuals (75%) were diagnosed with benign diseases. They determined that individuals with hyperthyroidism had a much greater chance of developing cancer relative to the total risk of the other two groups.

In this study, the majority (29, 72.5%) of the participants had swellings of sizes ranging from 2 to 4 cm. Nine (22.5%) had a swelling of size >4 cm, and two (5%) had a swelling of size <2 cm. The surface of the swelling was smooth in the case of 52.5% of the participants and nodular in the case of 47.5% of the participants. The lower border of the thyroid was palpable in all but one participant in this study. Melak et al. reported that the mean diameter of the thyroid swelling in their study was 6.1 cm, ranging from 1.13 cm to 19.54 cm [17]. They also observed that 96.9% of their study population had an enlargement of the thyroid which is 3.5 cm in diameter and more. Another study by Lo et al. reported that most tumors in their study had a size between 2 and 4 cm [5].

On palpation, the consistency of the thyroid swelling was found to be hard among 11 (27.5%) of the participants in this study. It was firm in the case of 25 (62.5%) and soft in the case of four (10%) of the subjects. The swelling was not mobile among the two (5%) of the subjects in this study. Lymph node enlargement was seen in 13 (32.5%) of the participants in this study. Firm consistency or enduring thyroid swelling, according to a review article by Eng et al., is indicative of an elevated likelihood of cancer [19]. Soft, mobile, or cystic swelling is indicative of low chances of cancer [19]. Prasanth et al. found that most of the thyroid nodules examined had a hard consistency [16]. Additionally, all the cases showed movement during swallowing and did not exhibit any enlargement of cervical lymph nodes. The study on WDTC by Lo et al. reported that 18% of their study population had palpable lymph nodes at presentation in their study [5].

Radiological assessment of the thyroid gland of participants in this study revealed that the majority (26, 65%) of the study population showed hypoechogenicity. Microcalcification was seen among 29 (72.5%) of the participants. Increased peripheral vascularity was observed in 31 (77.5%) of the subjects. This study also observed a loss of peripheral halo among 19 (47.5%) of the study population. The study on WDTC by Lo et al. reported that 76.4% of PTC cases and 77% of follicular thyroid cancer cases in their study population had hypoechogenicity [5]. They also observed calcification among 23.3% of PTC cases and 20.3% of follicular thyroid cancer patients. In this study, 85% of the participants had papillary carcinoma, 12.5% had medullary carcinoma, and the rest had follicular carcinoma. The study by Lo et al. also observed that among their study population, the majority (89%) had papillary carcinoma and only 11% had follicular thyroid carcinoma [5].

The histological type of thyroid cancer and the age range of the study population did not show any statistically significant association. Girardi et al. conducted a study that found a statistically significant correlation between the age category and the histological type of thyroid cancer [20]. The study also noted that papillary carcinoma was present in all age categories; however, it had a higher proportional incidence between the ages of 31 and 50. Within the older age cohorts, papillary carcinoma had a comparatively lower prevalence, while follicular, poorly differentiated, and anaplastic carcinoma were observed at a higher frequency. Furthermore, this study did not find any statistically significant correlation between gender and the histological type of thyroid cancer. Similar findings were noted by Freitag et al. who found no evidence of a substantial gender difference in terms of the histological type [21]. The study also indicates that age did not have an impact on histological characteristics among the men in the study. The female participants diagnosed with papillary and medullary cancer exhibited a statistically significant age difference compared to those diagnosed with follicular and anaplastic cancers.

In this study, the duration of the swelling was associated with the histological type of thyroid malignancy. In this study, a longer duration of the swelling was observed among medullary carcinoma and PTC. The study on WDTC by Lo et al. reported a higher mean duration of the thyroid swelling for follicular carcinoma cases compared to PTC cases [5]. The mean duration of the swelling was 65 ± 76 months for PTC cases and 77 ± 89 months for follicular carcinoma cases. In this study, there was no significant association between history of pain, pressure effect, and hoarseness of voice and histological type of thyroid malignancy. Eng et al. reported that the size of thyroid nodules directly correlates with compressive symptoms [19]. Additionally, they indicate that 97% of those with compressive symptoms and a thyroid nodule size greater than 1.5 cm achieved symptom alleviation after the surgical procedure.

In this study, there was no statistically significant association between the histological type of thyroid malignancy and the size or type of the thyroid swelling. A study on thyroid nodule size and prediction of cancer reported that there is a nonlinear increase in cancer risk as the size of thyroid nodules increases. Beyond 2 cm, cancer risk remains unchanged. However, the risk of follicular carcinoma and other rare types of thyroid malignancies increases as the size of the nodule increases [22]. Another study on the shape of thyroid nodules and the prediction of malignancy reports a correlation between the spherical shape of the thyroid swelling and the risk of malignancy with a p-value of <0.001. The study also found that 11% of all nodules were malignant, with spherical nodules having a higher rate of 18% and barely spherical nodules having a lower rate of 5% [23].

This study noticed a statistically significant association between the mobility of the swelling and the histological form of thyroid cancer. Thyroid swellings were not mobile in medullary carcinoma cases in this study. The type of thyroid cancer and lymph node presence were not associated with this investigation. The study on WDTC by Lo et al. reported that lymph node involvement was seen in 23% of PTC cases and 6.8% of follicular carcinoma cases in their study [5]. In this study, there was no statistically significant association between the histological type of thyroid malignancy and ultrasonographic findings of the swelling, like echogenicity, microcalcification, increased peripheral vascularity, or loss of peripheral halo. Lo et al. further reported that among the PTC cases, 76.4% had hypoechogenicity and 23.3% had microcalcifications [5]. The same study reports that among follicular carcinoma cases, 77% had hypoechogenicity and 20.3% had calcifications.

Limitations of the study include its retrospective design, which inherently relies on available medical records, potentially introducing selection bias. Additionally, the study was conducted at a single center, limiting the generalizability of the findings to other populations. The sample size, while meeting the calculated minimum, might still be relatively small for certain subgroup analyses. Furthermore, the reliance on medical records for data collection may have led to incomplete or inaccurate information, affecting the validity of the results. Lastly, the study's scope was limited to clinicopathological and radiological profiles, and factors such as genetic predispositions or environmental exposures were not explored, which could provide further insights into thyroid malignancies.

## Conclusions

The study did not uncover any statistically significant associations between age, gender, and the histological type of thyroid cancer. Clinical characteristics such as the duration of the swelling, soreness, pressure effects, hoarseness of voice, thyrotoxicosis symptoms, and hypothyroidism symptoms also showed no significant associations. Additionally, no significant correlations were found between the histological type of thyroid malignancy and the size, surface type, or consistency of the thyroid swelling. The presence of lymph nodes did not correlate with the type of thyroid cancer. Ultrasonographic characteristics such as swelling, echogenicity, microcalcification, increased peripheral vascularity, or loss of peripheral halo also did not show significant associations with histological type. However, the study did reveal that the duration of the swelling was linked to specific histological types of thyroid cancer, with medullary carcinoma and PTC showing longer durations of the swelling. Furthermore, a statistically significant correlation was found between the mobility of the swelling and histological type, with medullary carcinoma cases showing immobile swellings. While these findings provide insights, larger-scale studies with extensive sample sizes are warranted to robustly establish relationships between clinical symptoms, signs, and histological types of thyroid malignancies.
